# “Reduced Public Coverage Did Not Decrease Dental Visits”: Fact or Fiction?

**DOI:** 10.1177/00220345251332194

**Published:** 2025-05-12

**Authors:** Z. Sarroukh, P. Jeurissen, S. Listl

**Affiliations:** 1 Department of Dentistry, Quality and Safety of Oral Health Care, Radboud University Medical Center, Nijmegen, the Netherlands; 2 IQ Health, Radboud University Medical Center, Nijmegen, the Netherlands; 3 Heidelberg Institute of Global Health, Section for Oral Health, Heidelberg University Hospital, Heidelberg, Germany

**Keywords:** access to care, dental economics, dental insurance, health care reform, national health policy, universal coverage

## Abstract

Challenges of access have sparked political debates about dental coverage in the Netherlands. So far, policy makers have been opposing an expansion of public coverage for the adult population, arguing that previous reductions of public coverage would not have led to reduced dental attendance within the Dutch population. To this end, this study aims to evaluate the effects of public coverage reductions since 1995 on dental care utilization. We used a population-wide data set from Statistics Netherlands containing dental care utilization rates across insurance- and age-based subpopulations between 1981 and 2019. Drawing from an interrupted time series design with segmented regressions, we evaluated how scaling down public coverage for curative dentistry in 1995 and preventive dentistry in 2004 affected dental attendance. Population groups that were not subject to the reforms served as control groups (i.e., nonadult population and privately insured persons). Following the 1995 reform and in comparison with privately insured persons (control group), the dental attendance of publicly insured persons (intervention group) decreased by an immediate 3.5 percentage points (95% CI, −5.8 to −1.1) and by a subsequent 0.6 percentage points (95% CI, −0.9 to −0.3) per year. When compared with that of the nonadult population (control group), dental attendance by 20- to 45-y-olds (intervention group) immediately dropped by 4.6 percentage points (95% CI, −7.0 to −2.1) and subsequently by 1.2 percentage points (95% CI, −1.5 to −0.8) per year. Contrary to Dutch policy makers’ arguments, our findings suggest that reduced public coverage led to fewer people visiting the dentist in the Netherlands. These findings highlight the central role of policy evaluation for evidence-informed oral health policy making.

## Introduction

On average, 69% of total oral health expenditures in Europe are financed privately (Winkelmann et al. 2022). As costs of oral health care continue to rise, an ever-increasing share of the burden is carried by the private expenditures of patients. Subsequently, oral health care forms one of the main drivers of catastrophic health expenditures (Jevdjevic et al. 2020; World Health Organization 2023). Moreover, trends of rising costs have been accompanied by a substantial disease burden, especially among the poor. Cost barriers experienced due to limited oral health coverage have led to worldwide concerns of access to care. As a result, oral health care has gained traction on the global health agenda, with the World Health Assembly instating a vision for universal dental coverage for all by 2030 (World Health Organization 2023).

Similar concerns have been raised in the Netherlands, where coverage of oral health care is limited. Municipalities have seen a growing number of individuals foregoing dental care due to financial constraints (BS&F 2023). Begovic et al. (2023) find that costs form one of the main drivers of foregone dental care. Therefore, these trends have sparked political debates about a reuptake of oral health care in public coverage as part of the basic benefits package. Several political parties have shown interest in an expansion of coverage, while others expressed doubts regarding the effectiveness of such a reform (Van Gool and Van Noort 2023).

Several studies have presented evidence on the effects of changes in health coverage on the accessibility of general health care. Estimates provided by the RAND Health Insurance Experiment pointed toward an overall price elasticity of −0.2, suggesting moderate levels of moral hazard. Corresponding elasticities were found in the demand for dental care (Sintonen and Linnosmaa 2000). Elani et al. (2021) reported a 12.4–percentage point increase in dental visits as a result of expanded Medicaid coverage. Similarly, expansions of dental coverage in Finland were accompanied by a 4.2–percentage point increase in dental attendance (Raittio and Suominen 2022). Allen et al. (2023) noted that older adults in Singapore receiving subsidies for dental care were significantly more likely to attend regular dental visits as compared with adults not receiving this subsidy. While dental care subsidies in Sweden were shown to reduce overall unmet dental care needs, inequity in unmet needs appeared to have increased (Anticona et al. 2024). Moreover, private insurance in addition to public coverage was shown to increase dental visits in Catalonia (Pizarro et al. 2008).

Notably, the empirical literature has focused on increases in coverage of dental care, with limited acknowledgment of reduced coverage seen globally. Palència et al. (2014) reported a significant increase in socioeconomic inequality associated with European countries with lower levels of public coverage. Contrarily, Manski et al. (2016) found no significant association of dental care utilization to different levels of dental coverage across oral health care systems in Europe. These studies are based on somewhat older nonlongitudinal data. More recently, Vesinurm et al. (2023) noted a shift from utilization of dental care in the private sector to the public sector following substantial reductions of subsidies for private care. To our knowledge, there have been no previous studies on the effects of the removal of dental coverage from the basic benefits package on accessibility of oral health care in the Netherlands.

In light of the ongoing political debate, the Dutch Minister of Health opposed an expansion of public coverage for dental care, arguing that the proportion of adults who access oral health care has not changed due to the reductions of dental coverage since 1995 (Ministry of General Affairs 2022; Van Gool and Van Noort 2023; Vermeer 2023). Since the Second World War, coverage of health care in the Netherlands has been provided by sickness funds, voluntary insurance, and private insurance (Bertens and Vonk 2020; Jeurissen and Maarse 2021). Employees below a predetermined income threshold were required to take out insurance plans from the public coverage scheme of the sickness funds, which covered a uniform benefits package financed through employer and employee contributions. Unemployed individuals were excluded from this mandate, and those below the income threshold could subscribe to a voluntary scheme offered by the sickness funds. Employees above the income threshold had to opt for private insurance instead. Mandatory and voluntary schemes of sickness funds were heavily regulated by the government. In contrast, private insurers were largely free to design their own insurance products. In 1986, the voluntary scheme was abolished, and individuals had to opt for the mandatory sickness fund or private insurance, depending on their income level. These coverage schemes were mutually exclusive, as individuals were either subject to the mandate of the sickness funds or left to choose between private insurance and out-of-pocket payment. Eventually, in 2006, sickness funds and private insurance were merged into a private scheme with an individual mandate and open enrollment (Varkevisser and Van Der Geest 2003; Bertens and Vonk 2020; Jeurissen and Maarse 2021).

Rising health expenditures and budgetary constraints meant that the expenditure growth of the health care sector was restricted to 1.3% in the 1990s (House of Representatives of the Netherlands 1995). Hence, in the years following this report, several health care services were evaluated and removed from the benefits package based on disease burden, effectiveness, efficiency, and feasibility, as described by the Dunning committee. While dental care for children fulfilled all 4 criteria, services for adults were considered to be individuals’ own responsibility, leading to a reduction of dental coverage for citizens aged >17 y based on the fourth criterion (Post 2016). In 1995, the majority of dental care services were removed from coverage by the sickness funds, leaving only coverage for surgical, prosthetic, and preventive care. Coverage of preventive dentistry consisted of routine dental check-ups, scaling, and dental health advice (Statistics Netherlands 1998). However, coverage of preventive care was also removed for adults in 2004 (Poorterman and Schuller 2006).

Against this background, the aim of this study was to evaluate the empirical evidence underlying the argument that the proportion of adults who access oral health care has not changed due to reduced public coverage since 1995 through state-of-the-art empirical policy analysis on dental attendance.

## Materials and Methods

### Data

To study the effect of reduced dental coverage on dental visits, we used publicly available data from the health survey of Statistics Netherlands. This health survey collects data on health and health care utilization of the Dutch population through annual surveys since 1981, with representative population samples ranging between 8,442 and 11,117 individuals annually (National Institute for Public Health and the Environment 2024; Statistics Netherlands 2025).

We estimated the effects of the coverage reforms by comparing dental attendance of subpopulations contained in the data that were differentially affected by the reforms. Given that policies regarding the basic benefits package applied to the public coverage scheme, privately insured individuals were unaffected by the coverage changes of 1995 and 2004 until the sickness fund and private insurance were merged in 2006. Therefore, the publicly insured population served as an intervention group to compare with the privately insured population, which served as a control group, to identify differential dental attendance following reforms.

Similarly, individuals aged <18 y remained covered across both reforms, as the coverage reduction applied only to adults. Age-related subpopulations within the data facilitated comparisons between the 0- to 20-y-old cohort, a largely unaffected population serving as a control group, and the 20- to 45-y-old cohort, the intervention group. Contrary to curative and preventive dental care, dentures remained covered in the basic benefits package across the reforms, leading to potential bias in age-related comparative analyses. Therefore, age cohorts excluded the population with dentures.

### Dependent Variables

Our dependent variable is dental attendance, defined as the share of the population that visited a dentist. Participants of the health survey were asked annually if they had visited a dentist at least once in the past 12 mo. We analyzed dental attendance in the years before, between, and after 1995 and 2004, across the intervention and control groups. This gave us 14 y of observations before 1995 and 9 y of observations between the reforms for each subpopulation. Given that the sickness fund and private insurance merged in 2006, data points following the reduction in 2004 were limited to 2 y of observations for each insurance-based subpopulation. Dental attendance of the 0- to 20-y-old cohort and 20- to 45-y-old cohort was available up to the year 2009, providing 6 y of observations following 2004 reductions for each cohort (Statistics Netherlands 2010). The additional years of observations across age cohorts allowed us to capture changes in dental attendance after 2004.

### Covariates

Data on gross domestic product per capita and average household income were collected for robustness checks, as they indicate financial situations at the household and country levels (Appendix). These are relevant given that dental care is often considered a luxury good (Holden 2019; CPB Netherlands Bureau for Economic Policy Analysis 2024). Moreover, we assessed population composition across the reforms, including average age, proportion that is male, and proportion with a foreign background (Statistics Netherlands 2022).

### Statistical Analysis

This study adopted an interrupted time series analysis to estimate the effect of reduced coverage on dental visits. This design facilitates the evaluation of immediate and long-term effects of nonrandomized interventions through comparison of data before and after the reforms. Importantly, the interrupted time series analysis allows for estimation of effects through population-level data (Penfold and Zhang 2013). A theoretical model describing potential effects of coverage changes can be found in the Appendix.

To estimate the effect of reduced coverage, we specified the reduction in 1995 and 2004 in our regressors. We exploited the differential impact of the reforms on sickness fund–insured and privately insured subpopulations through a controlled interrupted time series (CITS) design. Similarly, differential impact across the populations aged 0 to 20 y and 20 to 45 y was estimated. We applied a Cumby-Huizinga test to measure autocorrelation and adjusted standard errors accordingly. This resulted in the following CITS model:

(1) $$\begin{array}{l} Y_{t}=\beta_{0}+\beta_{1}T_{t}+\beta_{2}X_{t}+\beta_{3}X_{t}T_{t}+\beta_{4}C_{t}\\ \;\;\;+\beta_{5}C_{t}X_{t}+\beta_{6}C_{t}T_{t}+\beta_{7}C_{t}T_{t}X_{t}+\beta_{8}P_{t}\\ \;\;\;+\beta_{9}P_{t}X_{t}+\beta_{10}P_{t}T_{t}+\beta_{11}P_{t}T_{t}X_{t}+\varepsilon_{t}. \end{array}$$

In this equation, *Y_t_* is a vector for the share of the population that has visited the dentist during year *t*. *T_t_* denotes time in years capturing secular trends. *X_t_* is an indicator for the intervention group, representing publicly insured and adult populations. *C_t_* and *P_t_* represent dummy variables for the reduction of curative and preventive dentistry, respectively, taking value 1 postreform and 0 otherwise. *TC_t_* and *TP_t_* denote years since reduced coverage for curative and preventive dentistry. Indicator *X_t_* was interacted with all variables to estimate differential impacts between the intervention and control groups. The parameters of interest β_5_ and β_9_ show the immediate differential change between the intervention and control groups due to reduced coverage, while β_7_ and β_11_ show the sustained change in dental visits.

Given that the dependent variable is bounded between 0% and 100%, we checked robustness against nonlinearity by estimating a logit model and compared predicted dental attendance vectors between the models. Additionally, we estimated an interrupted time series of the overall population between 1981 and 2019, controlling for average household income and gross domestic product per capita. Data on annual dental attendance rates across 0- to 20-y-olds, 20- to 45-y-olds, 45- to 65-y-olds, and adults aged ≥65 y were used to add cohort fixed effects. These robustness checks can be found in the Appendix.

All analyses were conducted through Stata version 18 (StataCorp). The itsa and xtitsa packages were used to perform the analyses (Linden 2015, 2021). The study did not require a review from the Dutch Medical Ethical Clearance Commission and the ethics commission at Radboud University Medical Center, given that participants were not subjected to actions that fall under the Medical Research Involving Human Subjects Act.

## Results

Table 1 presents summary statistics with average values of the Dutch population composition and dental attendance across the periods before, in between, and after dental coverage reduction (Statistics Netherlands 2022). A careful examination of the time series data for these demographic characteristics between 1981 and 2019 reveals no particular shocks or changes in trends interfering with the removal of coverage for dentistry. Therefore, we find no reason to assume that the reforms in 1995 and 2004 are endogenous to changes in population composition. A change in trend can be noted in the proportion of the population that visits the dentist. The average dental care utilization increased by 8.3 percentage points across the years before and after removal of curative dentistry. In contrast, the proportion visiting the dentist increased by 1.8 percentage points during the years across removal of preventive dentistry.

**Table 1.** Average Population Composition Pre- and Postreform.

		Descaling of Coverage	
	Prereform Period, 1981 to 1994	For Curative Dentistry, 1995 to 2003	For Preventive Dentistry, 2004 to 2019
**Demographics**			
Population size, *n*	14,711,431	15,783,157	16,696,347
Male, %	49.5	49.5	49.5
Mean age, y	36.1	38.0	40.4
Background, %^a^			
Migration	—	17.4	20.9
Non-Western migration	—	8.6	9.3
**Dependent variable**			
Dental visits, %	68.6	76.9	78.7

Statistics were processed with data from Statistics Netherlands (2022, 2023).

a Data were unavailable for the 1981–1994 period.

Estimates of the interrupted time series analyses are reported in Table 2. Dental visits of the sickness fund–insured population decreased by an immediate 3.5 percentage points (95% CI, −5.8 to −1.1) and a sustained 0.6 percentage points (95% CI, −0.9 to −0.3) as compared with the privately insured population. Similarly, dental visits among 20- to 45-y-olds decreased initially by 4.6 percentage points (95% CI, −7.0 to −2.1) as compared with 0- to 20-y-olds, followed by a reduction of 1.2 percentage points each year (95% CI, −1.5 to −0.8). However, limited evidence was found for significant effects following the reduction of coverage in 2004. Robustness checks for various model specifications produced similar results (Appendix).

**Table 2.** Estimates for the Effect of Reduced Dental Coverage on Dental Attendance.^a^

	Interrupted Time Series, % (95% CI)	
	Insurance Controlled	Age Controlled
Control group		
Prereform trend	0.4 (0.3 to 0.6)	0.0 (0.0 to 0.1)
Level change 1995	2.2 (0.1 to 4.3)	1.4 (0.5 to 2.4)
Post-1995 trend	0.1 (−0.1 to 0.3)	0.1 (−0.1 to 0.3)
Level change 2004	−0.5 (−1.5 to 0.5)	−1.1 (−2.3 to 0.0)
Post-2004 trend	−1.9 (−2.1 to −1.7)	−0.2 (−0.4 to 0.1)
Intervention group^b^		
Prereform trend	0.6 (0.4 to 0.8)	0.9 (0.6 to 1.1)
Level change 1995	−3.5 (−5.8 to −1.1)	−4.6 (−7.0 to −2.1)
Post-1995 trend	−0.6 (−0.9 to −0.3)	−1.2 (−1.5 to −0.8)
Level change 2004	−1.0 (−2.5 to 0.5)	0.5 (−0.9 to 1.9)
Post-2004 trend	1.3 (1.1 to 1.6)	−0.2 (−0.6 to 0.1)

a Model based on annually collected data for the years 1981 to 2009.

b Coefficients of variables for intervention group represent relative change in level or trend compared to control group.

The Figure presents the predicted proportion of the overall Dutch population visiting the dentist between 1981 and 2009, excluding the population with dentures and adjusted for cohort fixed effects. The graph visualizes the changes in trend following reforms. While a consistent increase in dental visits could be noted before the reduction of coverage, this trend appears to stabilize substantially post-1995 and becomes negative after 2004.

**Figure.**
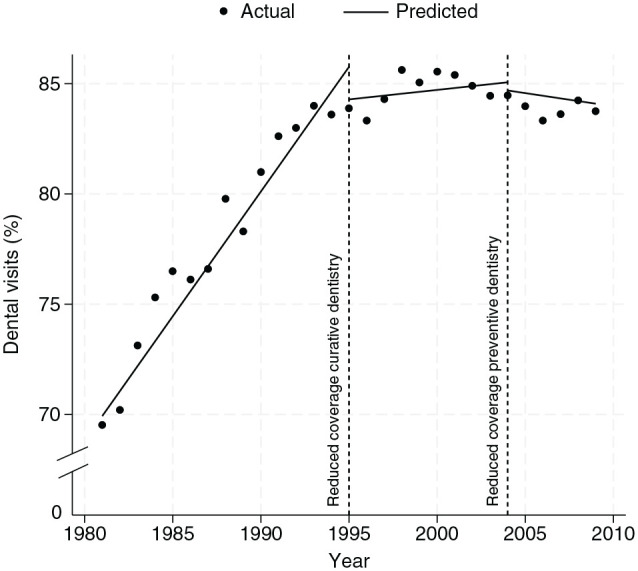
Predicted share of the population with at least 1 dental visit.

## Discussion

Concerns of foregone dental care resulting from the limited oral health coverage in the Netherlands have spurred political debates about the reuptake of oral health care into the basic benefits package. The Ministry of Health has recently held a stance against such reform based on the argument that dental attendance has not changed due to the reductions of coverage since 1995. The findings of this study suggest otherwise.

Oral health coverage in the Netherlands underwent a series of reductions since 1995. We find strong evidence to believe that the changes in 1995 are associated with an immediate as well as sustained reduction of dental visits among the population. Patients facing higher incurred prices could be reducing the demand for dental care as they trade off consumption of dental care with other goods, in line with theories of demand for dental care (Appendix).

Contrarily, limited evidence was found for the effects of the reduction of preventive care coverage in 2004. This may have been the result of an increase in supplementary insurance for dental care once public coverage was scaled down in 1995. Such market responses could protect patients against changes in incurred price due to reforms in 2004. Moreover, empirical evidence suggests various socioeconomic factors as strong determinants of dental attendance (Tu et al. 2023). Individuals with lower socioeconomic status may have been foregoing routine checkups before reforms, resulting in no change post-2004. Those with higher socioeconomic status, who were more likely to utilize regular dental checkups and were able to compensate for the reduction of coverage through out-of-pocket financing or supplementary insurance, would also not be affected.

These findings are consistent with several previous studies (Pizarro et al. 2008; Elani et al. 2021; Raittio and Suominen 2022; Allen et al. 2023; Vesinurm et al. 2023). Note that some earlier studies relied on older data sets with shorter observation periods, which may have led to differing findings (Pizarro et al. 2008; Palència et al. 2014; Manski et al. 2016). Differences in magnitudes among studies might reflect contextual and cultural differences among various health systems. Larger effect sizes as reported in previous literature (e.g., Elani et al. 2021) might stem from a different directionality of coverage change—that is, increased instead of reduced coverage. Coverage reductions might trigger provider concerns of losing revenue and behavior to compensate for such expected losses. The different effect sizes that we find when compared with previous literature might provide evidence for such responses.

Yet, patients could respond to reduced coverage through the uptake of supplementary insurance. Similar to the supply-side responses, this could negate some of the effects of reduced coverage. Importantly, overall uptake of supplementary insurance has gradually decreased in the Netherlands since 2006 (Vektis 2024). Such changes to supplementary insurance during our study period may have contributed to the estimated long-term effect of coverage reductions, given that changes in uptake likely coincided with the reforms. Nevertheless, we find that the policy shocks, especially since 1995, have resulted in fewer dental visits.

This research poses the first empirical study on the effects of the reduction of dental coverage in the Netherlands in 1995 and once again in 2004. Given the time horizon of this study, we were able to capture long-term effects after market adjustments to reforms. Applying CITS and secondary analyses, we find a reduction of dental visits after removal of coverage. However, potential limitations were encountered that should be addressed in light of our conclusions. The data set used in this study consists of aggregated macrolevel data based on the statistics published by Statistics Netherlands. A limited number of data points were available after the reduction of coverage in 2004, especially for the insurance-based CITS model. Therefore, a careful interpretation of the effects of policy changes in 2004 in this model is warranted. Moreover, interpretation of our estimates is limited to aggregate population and health system responses. Given that the dependent variable is bounded between 0% and 100%, a flattening in the trend of dental attendance would likely occur below 100% regardless of the coverage reforms. While the relationship with the dependent variable is therefore nonlinear, Angrist and Pischke (2008) and Wooldridge (2001) have shown that linear models, as estimated in this article, are able to approximate the underlying average marginal effects of the intervention. The validity of our findings is corroborated by robustness checks.

As political attention for oral health care continues to rise, empirical evidence meeting windows of opportunity becomes increasingly important to prevent biases in policy and erroneous decisions. While those opposed to the reuptake of oral health care into the basic benefits package in the Netherlands argue that utilization rates did not change due to reduced public coverage, we find evidence against this claim. These results imply potential arguments in favor of dental care reuptake into public coverage. More generally, these findings highlight the central role of policy evaluation for evidence-informed oral health policy making (Listl et al. 2023). Similarities in oral health policy-making processes and in public dental coverage across European countries, such as Denmark, the Netherlands, and the United Kingdom, suggest a shared relevance of evidence on coverage reforms. Arguments presented in this study could serve to debunk unsubstantiated policy-making narratives for shifting public responsibilities to the private sector (Sarroukh et al. 2024). Given that the Netherlands has one of the highest dental attendance rates in Europe, many countries have greater potential to raise dental attendance before reaching saturation (Winkelmann et al. 2022). Countries that have maintained limited dental coverage may have imposed greater restrictions on improvements in dental attendance. Therefore, our estimates could serve as a reference, providing a lower bound on the effect of the reduction of coverage on dental care utilization.

## Author Contributions

Z. Sarroukh, contributed to conception, design, data acquisition, analysis, and interpretation, drafted and critically revised the manuscript; P. Jeurissen, contributed to conception, design, data interpretation, critically revised the manuscript; S. Listl, contributed to conception, design, data acquisition, analysis and interpretation, critically revised the manuscript. All authors gave final approval and agree to be accountable for all aspects of the work.

## Supplemental Material

sj-docx-1-jdr-10.1177_00220345251332194 – Supplemental material for “Reduced Public Coverage Did Not Decrease Dental Visits”: Fact or Fiction? Supplemental material, sj-docx-1-jdr-10.1177_00220345251332194 for “Reduced Public Coverage Did Not Decrease Dental Visits”: Fact or Fiction? by Z. Sarroukh, P. Jeurissen and S. Listl in Journal of Dental Research
