# Supplementary material for: “Reduced Public Coverage Did Not Decrease Dental Visits”: Fact or Fiction?
Source: J Dent Res. 2025 May 12;104(11):1202–7. doi: 10.1177/00220345251332194 (PMC12426323; doi:10.1177/00220345251332194)
Supplement: sj-docx-1-jdr-10.1177_00220345251332194 – Supplemental material for “Reduced Public Coverage Did Not Decrease Dental Visits”: Fact or Fiction? [file sj-docx-1-jdr-10.1177_00220345251332194.docx]

**Appendix for article titled: ‘“Reduced Public Coverage Did Not Decrease Dental Visits”: Fact or Fiction?’**

Ziade Sarroukh^1^, Patrick Jeurissen^2^, Stefan Listl^1, 3^

^1^Department of Dentistry, Quality and Safety of Oral Health Care, Radboud University Medical Center, Nijmegen, The Netherlands

^2^IQ Health, Radboud University Medical Center, Nijmegen, The Netherlands

^3^Heidelberg Institute of Global Health, Section for Oral Health, Heidelberg University Hospital, Heidelberg, Germany

**An economic model of demand for dental care**

Insights on potential utilization of oral health care following changes to incurred price due to coverage removal can be derived from theories of utility maximization. A simplified model focused on price effects due to coverage change is described in this section, following established theoretical concepts (Hay et al. 1982; Holtmann and Olsen 1976; Sintonen and Linnosmaa 2000; Zweifel et al. 2009). Suppose patient utility is a function of oral health and consumption of other goods:

(1)

$$U\left( D, C \right)$$

where $D=D(z)$. Importantly, oral health $D$ is increasing in dental care $z$, as dental care contributes to oral health of patients. Moreover, utility is increasing in oral health and other consumption. Depending on the extent of coverage for oral health care in health systems, patients must pay price $p_{z}$ to consume dental care. However, budgetary constraints conditional on income limit consumer expenditures on health and other consumption:

(2)

$$p_{z}z+ p_{C}C=I$$

Substituting the budget constraint into the utility function yields:

(3)

$$Q\left( z \right)=(D\left( z \right), \frac{I- p_{z}z}{p_{C}})$$

where we deduce the first order condition $\frac{\partial Q}{\partial z} = 0$ as:

(4)

$$\frac{\partial U}{\partial D}\frac{\partial D}{\partial z}-\frac{\partial U}{\partial C}\frac{p_{z}}{p_{c}} = 0$$

with second order condition $\frac{\partial^{2}Q}{\partial z^{2}} < 0$:

(5)

$$\frac{\partial^{2}U}{\partial D^{2}}\frac{{\partial D}^{2}}{\partial z} + \frac{\partial U}{\partial D}\frac{\partial^{2}D}{\partial z^{2}} + \frac{\partial^{2}U}{\partial C^{2}}\frac{{p_{z}}^{2}}{{p_{C}}^{2}} < 0$$

assuming diminishing marginal utility of oral health and other consumption, and diminishing returns of dental care in oral health. Eq. 4 implies that a utility-maximizing consumer chooses an amount of dental care where the marginal benefit equals the marginal cost. To determine the effect of increased price due to coverage removal on dental care utilization, we apply implicit function theorem. A total differentiation of the first order condition yields:

$$\frac{\partial^{2}Q}{\partial z^{2}}dz + \frac{\partial^{2}Q}{\partial p_{z}\partial z}dp_{z} = 0$$

where the expression $\frac{\partial^{2}Q}{\partial p_{z}\partial z}$ denotes the cross partial derivative of dental care utilization with respect to price of dental care. Based on Eq. 4, we deduce that this expression consists of:

(6)

(7)

$$\frac{\partial^{2}Q}{\partial p_{z}\partial z} = -\left( \frac{\partial U}{\partial C} \right) + \frac{\partial^{2}U}{\partial C^{2}}\frac{zp_{z}}{p_{C}}$$

so that:

(8)

$$\frac{dz}{dp_{z}} = \frac{-{\partial^{2}Q}/{\partial p_{z}\partial z}}{{\partial^{2}Q}/{\partial z^{2}}} = \frac{\frac{\partial U}{\partial C}- \frac{\partial^{2}U}{\partial C^{2}}\frac{zp_{z}}{p_{C}}}{\frac{\partial^{2}U}{\partial D^{2}}\frac{{\partial D}^{2}}{\partial z} + \frac{\partial U}{\partial D}\frac{\partial^{2}D}{\partial z^{2}} + \frac{\partial^{2}U}{\partial C^{2}}\frac{{p_{z}}^{2}}{{p_{C}}^{2}}} < 0$$

Given that $\frac{\partial^{2}Q}{\partial z^{2}}<0$, $\frac{\partial^{2}U}{\partial C^{2}}<0$ and $\frac{\partial U}{\partial C}>0$, we find that $\frac{dz}{dp_{z}}<0$. Hence, Eq. 8 shows that dental care utilization is decreasing with increasing price.

Given the limited availability of microlevel data, we were unable to study the effects of coverage on individual patient behaviour and resulting price elasticities. Instead, we focus on changes in equilibrium resulting from aggregated shifts in patient behaviour using macrolevel data. Appendix Figure 1 illustrates potential changes, where line D represents the demand for dental care prior to the reduction of dental coverage. Reforms in 1995 and 2004 could raise private financing of patients, resulting in an inward shift of the demand curve, illustrated with line D’.

Since the reduction of dental coverage was a nation-wide policy for the sickness fund insured population, aggregated implications could be substantial and subsequent health system responses are likely (Stoddart et al. 1993). The market for supplementary insurance of dental care could become more enticing for both patients and insurers. Uptake of supplementary insurance would negate some of the effects as patients are once again (partially) financially protected against costs of dental care. Hence, an outward shift of the demand curve could be observed. Moreover, dental care providers could experience large reductions in income, to which dentists may respond through supplier induced demand. Dentists could promote dental visits for patients to maintain demand, in turn shifting the demand curve outwards alongside the supply curve (Stoddart et al. 1993; Van De Voorde et al. 2006). These responses would lead to a new long-term equilibrium alongside line D’’.

**Appendix Figure 1.** Demand for dental care


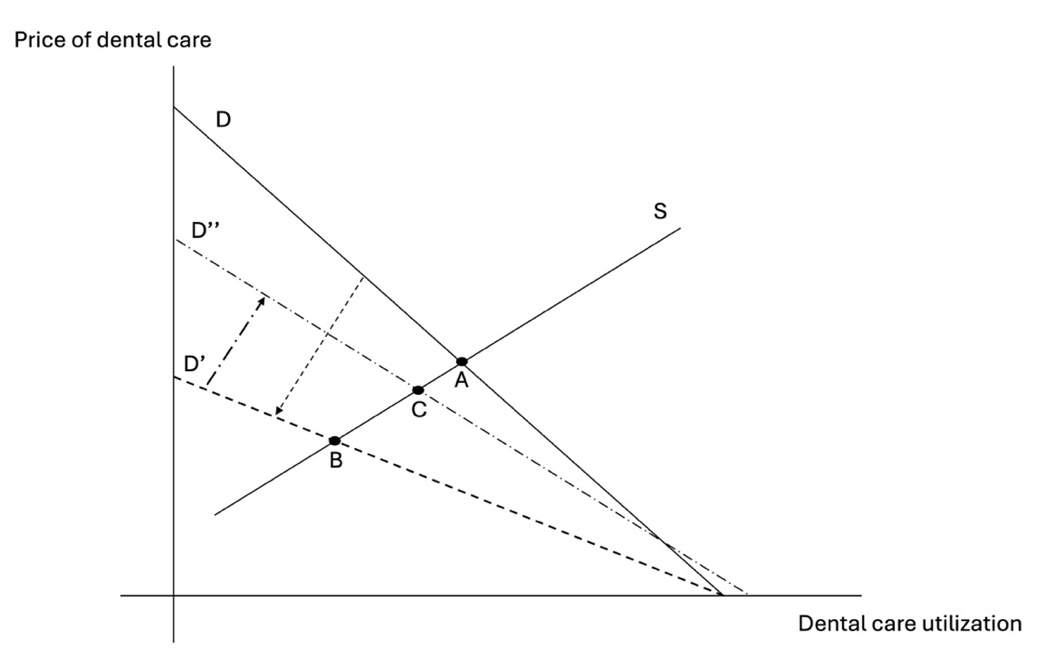


Most of the empirical evidence has focused on isolating the effect of costs on individual patient demand, with the RAND Health Insurance Experiment remaining one of the most prominent to date. An important limitation of these studies is the inability to capture broader health system responses to the price of health care, such as supplier induced demand as described above (Stoddart et al. 1993; Van De Voorde et al. 2006). Such effects are important for policymakers to grasp wider implications of interventions. While we are unable to distinguish between demand and supply effects, our aggregated dataset facilitates insights into long-term overall health system response.

**Robustness checks**

We estimated the insurance-based and age-based controlled interrupted time series using a logit model to check the robustness of our results against nonlinearities in model specification. The correlation between predicted dental attendance vectors of the linear and logit models can be found in Appendix Table 1. We find a strong correlation between the vectors, indicating robustness of our results against model specification.

Effects on the overall population were estimated through a single group ITS. Again, both the reduction in 1995 and 2004 were specified in our regressors (Wagner et al. 2002). Given that cost considerations may have played a role in the reduction of coverage, we accounted for GDP per capita and average household income between 1981 and 2019 (CPB Netherlands Bureau for Economic Policy Analysis 2024). A Cumby-Huizinga test was used to measure autocorrelation, after which standard errors were adjusted accordingly (Schuch et al. 2024). This resulted in the following single group ITS model:

(9)

$$Y_{t}= \beta_{0} + \beta_{1}T_{t} + \beta_{2}C_{t} + \beta_{3}T_{t}C_{t} + \beta_{4}P_{t} + \beta_{5}T_{t}P_{t} + \beta_{6}{GDP}_{t} + \beta_{7}{HHI}_{t} + \varepsilon_{t}$$

where $Y_{t}$ is a vector for the share of the population that has visited the dentist during year $t$. $T_{t}$ denotes time in years capturing secular trends. $C_{t}$ and $P_{t}$ represent dummy variables for the reduction of curative and preventive dentistry, respectively, taking value 1 postreform and 0 otherwise. ${TC}_{t}$ and $TP_{t}$ denote years since reduced coverage for curative and preventive dentistry. ${GDP}_{t}$ and ${HHI}_{t}$ denote our economic performance indicators. The parameters of interest $\beta_{2}$ and $\beta_{4}$ provide estimates for immediate change as a result of reduced coverage. Sustained changes in trends following reforms are captured by $\beta_{3}$ and $\beta_{5}$.

The repeated annual observations across the age categories 0-20-year-olds, 20-45-year-olds, 45-65-year-olds and adults aged 65 or older allowed us to exploit variation of dental visits within these cohorts, removing time invariant sources of bias through cohort fixed effects. This gave rise to the following estimated equation:

$$Y_{it}= \beta_{0} + \beta_{1}T_{it} + \beta_{2}C_{it} + \beta_{3}T_{it}C_{it} + \beta_{4}P_{it} + \beta_{5}T_{it}P_{it} +\Omega cohort+ \varepsilon_{it}$$

(10)

Estimates of the robustness checks can be found in Appendix Table 2. The single group ITS shows that the reduction of coverage in 1995 was followed by a 0.9 percentage points (95% CI, -1.7 to 0.0) decrease in the share of the population visiting a dentist. Moreover, the rising trend of dental visits before 1995 was decreased by 0.5 percentage points (95% CI, -0.7 to -0.2) every year after the reform. The removal of coverage for preventive dental care in 2004 was associated with an immediate 1.8 percentage points (95% CI, -3.0 to -0.6) reduction in dental visits, followed by a sustained 0.3 percentage points (95% CI, -0.6 to -0.1) reduction each year. Adding fixed effects to the ITS reveals an insignificant immediate impact of the 1995 reforms, but a significant sustained reduction of 1.0 percentage points (95% CI, -1.8 to -0.2) per year. No significant effects were found for the reform in 2004.

**Appendix Table 1.** Correlation between predicted dental attendance vectors of linear and nonlinear interrupted time series

|  | Vectors insurance controlled interrupted time series | Vectors age controlled interrupted time series |
| --- | --- | --- |
| Correlation | 1.0000 | 0.9986 |

**Appendix Table 2.** Estimates from models applying single group interrupted time series and cohort fixed effects

|  | Single interrupted time series, % | Cohort fixed effects, % |
| --- | --- | --- |
| Prereform trend | 0.9 (0.7; 1.1) | 1.1 (0.7; 1.5) |
| Level change 1995 | -0.9  (-1.7; 0.0) | -1.5 (-3.8; 0.9) |
| Post 1995 trend | -0.5 (-0.7; -0.2) | -1.0 (-1.8; -0.2) |
| Level change 2004 | -1.8 (-3.0; -0.6) | -0.4 (-2.8; 2.1) |
| Post 2004 trend | -0.3 (-0.6; -0.1) | -0.2 (-1.3; 0.9) |

*Note: 95% confidence intervals in parentheses*

**Appendix references**

CPB Netherlands Bureau for Economic Policy Analysis. 2024. CPB projections: purchasing power restored, public finances need a course correction. The Hague: CPB [accessed 2025 March 19]. https://www.cpb.nl/raming-februari-2024-cep-2024. Article in Dutch.

Hay JW, Bailit H, Chiriboga DA. 1982. The demand for dental health. Soc Sci Med. 16(13):1285–1289. https://doi.org/10.1016/0277-9536(82)90072-7.

Holtmann AG, Olsen EO Jr. 1976. The Demand for Dental Care: A study of Consumption and Household production. J Hum Resour. 11(4):546. https://doi.org/10.2307/145431.

Schuch HS, Furtado M, Filho ADPC, Elani HW. 2024. Changes in use of prenatal dental care after Brazil’s incentive policy. J Dent Res. https://doi.org/10.1177/00220345241258459.

Sintonen H, Linnosmaa I. 2000. Chapter 24 Economics of dental services. In Handbook of health economics (pp. 1251–1296). https://doi.org/10.1016/s1574-0064(00)80037-2

Stoddart, G. L., Barer, M. L., & Evans, R. G. 1993. User charges, snares and delusions: another look at the literature. RePEc: Research Papers in Economics. https://doi.org/10.14288/1.0048384

Van De Voorde C, Van Doorslaer E, Schokkaert E. 2001. Effects of cost sharing on physician utilization under favourable conditions for supplier‐induced demand. Health Econ. 10(5):457–471. https://doi.org/10.1002/hec.631.

Wagner AK, Soumerai SB, Zhang F, Ross‐Degnan D. 2002. Segmented regression analysis of interrupted time series studies in medication use research. J Clin Pharm Ther. 27(4): 299–309. https://doi.org/10.1046/j.1365-2710.2002.00430.x

Zweifel P, Breyer F, Kifmann M. 2009. Health Economics. Springer-Verlag Berlin Heidelberg. https://doi.org/10.1007/978-3-540-68540-1.
